# COVID-19 infections in German long-term care facilities: a descriptive three-level analysis using claims and infection statistics data from October 2020 to March 2021

**DOI:** 10.1186/s12889-026-26510-5

**Published:** 2026-02-07

**Authors:** Raphael Kohl, Kathrin Jürchott, Christian Hering, Annabell Gangnus, Elisabeth Steinhagen-Thiessen, Jan Paul Heisig, Adelheid Kuhlmey, Antje Schwinger, Paul Gellert

**Affiliations:** 1 Institute of Medical Sociology and Rehabilitation Science, Charité – Universitätsmedizin Berlin, corporate member of Freie Universität Berlin and Humboldt-Universität zu Berlin, Charitéplatz 1, 10117 Berlin, Germany; 2 WIdO – AOK Research Institute, Berlin, Germany; 3 Deparment of Endocrinology, Diabetes and Metabolism, Charité – Universitätsmedizin Berlin, corporate member of Freie Universität Berlin and Humboldt-Universität zu Berlin, Charitéplatz 1, 10117 Berlin, Germany; 4 WZB Berlin Social Science Center, Berlin, Germany; 5 Freie Universität Berlin, Berlin, Germany; 6 Einstein Center Population Diversity, Berlin, Germany

**Keywords:** COVID-19, SARS-CoV-2, Long-term care facilities, Nursing homes, Contextual effects

## Abstract

**Background:**

Although many studies have investigated COVID-19 outbreaks in long-term care facilities (LTCFs), evidence that combines multiple clustered levels is scarce. We aimed to describe individual, LTCF, and regional-level factors associated with COVID-19 infections.

**Methods:**

We conducted a nationwide study using insurance claims data from Germany between 1st October 2020 and 31st March 2021. The sample comprised 284,186 residents over 60 years in 9,869 LTCFs across all of Germany’s 400 districts. We used multilevel logistic regression to model associations between individual, LTCF, and district-level factors, and the probability of a COVID-19 infection.

**Results:**

A total of 44,042 (15.5%) COVID-19 infections were recorded during the study period. On the individual level, male sex (OR 1.15; 95% CI 1.12–1.18), dementia (OR 1.09; CI 1.06–1.11), medium-severe care dependency level 3 and 4 (OR 1.17; CI 1.12–1.22 / OR 1.21; CI 1.16–1.26) were associated with greater risk of infection. At the LTCF level, infection risks increased with the mean age of residents (OR 1.09; CI 1.03–1.15) and higher resident numbers (OR 1.20; CI 1.14–1.27). On the district level, a higher proportion of public LTCFs was associated with lower infection risks (OR 0.90; CI 0.84–0.97), while a higher mean number of residents (OR 1.16; CI 1.05–1.28), and the district-level SARS-CoV-2 incidence rate among the general population (OR 1.54; CI 1.41–1.67) was associated with higher risks. A cross-level interaction between facility size and COVID-19 prevalence was not significant (*p* > 0.5).

**Conclusion:**

We found evidence of individual, facility, and regional levels factors associated with COVID-19 infections among older adults in LTCFs. Future measures to combat infections, outbreaks, and pandemics should take an orchestrated multilevel approach.

**Supplementary Information:**

The online version contains supplementary material available at 10.1186/s12889-026-26510-5.

## Introduction

In the early months of the COVID-19 pandemic, it was already known that long-term care facilities (LTCFs) were particularly affected as they provided an excellent environment for an airborne virus to spread among a highly vulnerable population. Hence, already on 21 st March 2020, the WHO provided guidance for prevention and control for long-term care facilities. Those guidelines included staff training, the physical distancing of residents from one another and visitors, and the recognition and isolation of infected residents [1]. 

Despite these precautions, LTCF residents were disproportionally affected by the pandemic: In Germany, where fewer than 1% of the population lives in LTCFs, LTCF residents accounted for 2.3% to 4.0% of all COVID-19 cases registered by 31 st March 2021 [2, 3]. Nevertheless, we found only two studies which investigated COVID-19 outbreaks in German LTCFs [4, 5]. One study was based on data from the official national COVID-19 statistics which provide only basic information on individuals (i.e. age and sex), and none at all on the characteristics of LTCFs [4]. The second study relied on self-report questionnaires sent to LTCFs. These data contain information about facility-level infection rates and countermeasures but none about individual residents [5]. Hence, both studies leave out important aspects due to data limitations. A systematic review by Karimi-Dehkordi et al. gathered 99 studies on COVID-19 outbreaks and mortality in LTCFs and systematized them using a multilevel framework [6]. The individual studies included provided evidence for one or more of the following levels: (1) sociodemographic and condition-specific factors at the individual level, (2) LTCFs quality indications, staffing, ownership, Medicare and Medicaid coverage, and LTCFs racial and ethnic composition at the organizational level, and (3) community factors and characteristics of the physical conditions at the environmental level. The authors found that, at the individual level, infections were associated with older age, increased dependency, female sex, higher frailty, presence of comorbidities, and a cognitive decline/dementia. At the organizational level, infection rates were found to be related to staffing levels, facility ratings, the percentage of ethnic and racial minorities in LTCFs, ownership, and the presence of infected staff. Finally, at the environmental level, size and crowding, the regional incidence rate, community sociodemographic, and population density were the most important factors associated with COVID-19 infections among residents. While the systematic review by Karimi-Dehkordi et al. [6] provided an overview of relevant factors at various levels, evidence is still lacking from studies that examined individual, facility, and regional levels simultaneously and went beyond self-report or convenience data. By addressing this gap, this study aims to describe individual, facility, and regional factors contributing to COVID-19 infections in German LTCFs. It focuses on their impact during the second wave of the pandemic after pandemic measures were in place.

**Fig. 1:**
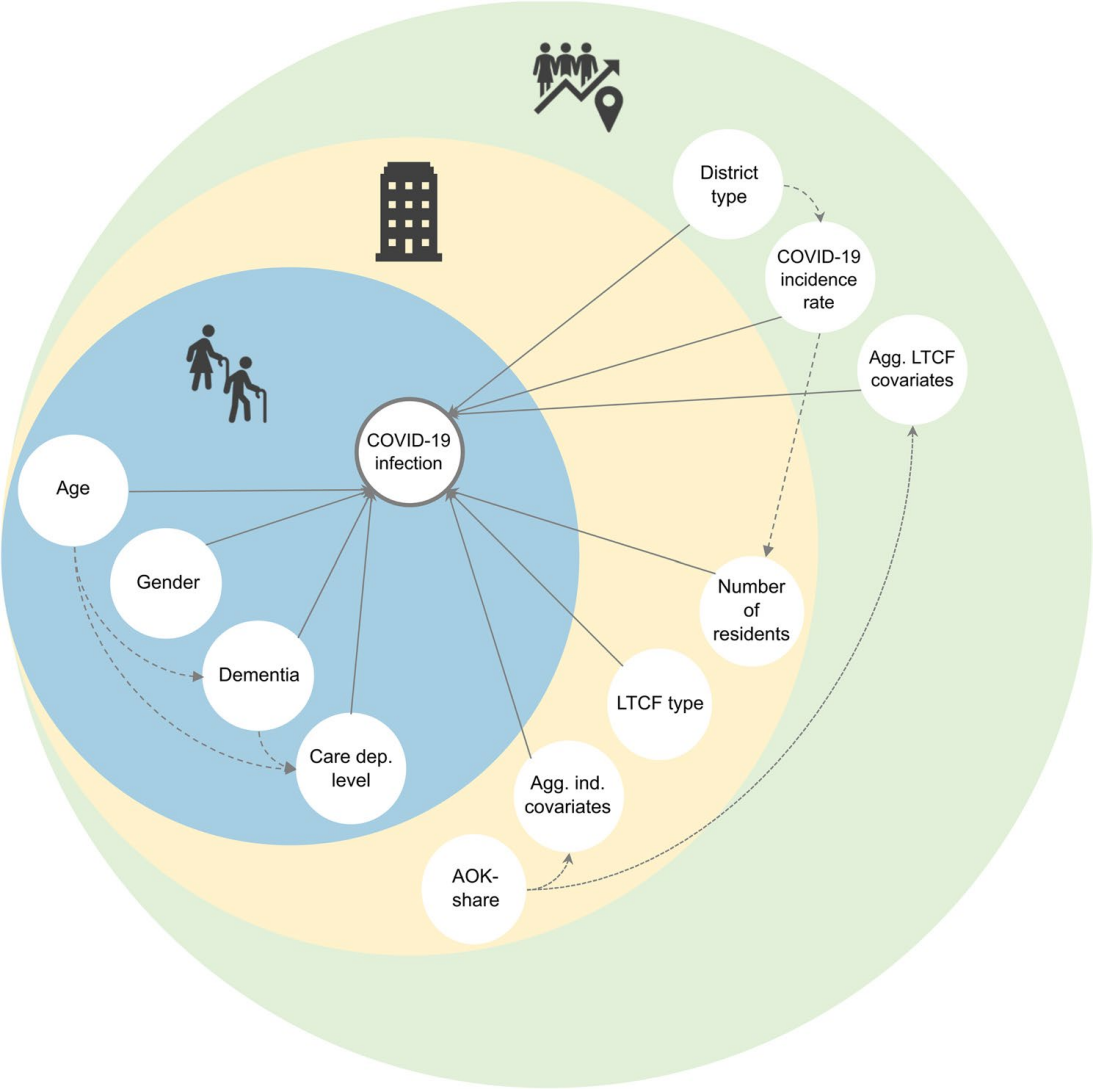
Proposed conceptual framework for the multilevel analysis of COVID-19 infections in long-term care facilities in Germany. Solid lines represent covariates of interest, dotted lines indicate potential confounders or factors affecting data quality, and dashed lines depict potential indirect causal pathways

As illustrated in our conceptual framework (Fig. 1), we examine how individual-level, facility-level, and district-/community-level factors, as generally proposed by the socio-ecological model, are assumed to be associated with COVID-19 infection risks among LTCF residents in Germany. Our analysis is based on claims data from the largest statutory health and long-term care insurance fund in Germany and observed mainly the second wave of the pandemic.

## Methods

### Study design and population

This retrospective observational study is based on claims data from the largest German statutory health and long-term care insurance fund (AOK). Germany has a dual health insurance system with statutory and private health insurances. In 2020 about 88% of the German population was insured with a statutory health insurance [7]. According to AOK´s own reporting, in 2020 AOK covered 46.6% of the statutorily insured residents living in LTCFs [8]. These LTCFs are run by non-profit, public or for-profit organizations. This study comprises all AOK-insured individuals who resided in a LTCF for at least one day during the observation period between 1 st October 2020 and 31 st March 2021. This observation period was aligned with the availability of the data, as information on COVID-19 diagnosis was only available by quarters. Thus, the observation period includes most of the second wave of the pandemic in Germany, which took place between 28th September 2020 and 28th February 2021, and the first half of the subsequent third wave from 1 st March 2021 to 13th June 2021 [9]. We analyzed data from 284,186 LTCF residents aged 60 years and older (Table 1) in 9,869 LTCFs (Table 2) and all 400 German districts (Table 3). COVID-19 infection data were obtained from the Robert Koch Institute (RKI) [10], while district-level characteristics were sourced from the Federal Institute for Research on Building, Urban Affairs and Spatial Development (BBSR) [11]. These datasets were matched to the claims data at the district level to enable a multilevel analysis.

**Table 1:** Descriptives level 1: LTCF residents (*N* = 284186)

		COVID-19 Infection		Total	SMD (95% CI)
		no	yes		
Gender	women	172,779 (84.8%)	30,976 (15.2%)	203,755 (71.7%)	0.04 (0.03, 0.05)
	men	67,365 (83.8%)	13,066 (16.2%)	80,431 (28.3%)	
Age	mean (SD)	84.1 (9.0)	84.4 (8.4)	84.2 (8.9)	−0.04 (−0.05, −0.03)
Care level	≤ 2nd level	33,874 (86.5%)	5268 (13.5%)	39,142 (13.8%)	0.09 (0.08, 0.10)
	3rd level	77,351 (84.3%)	14,438 (15.7%)	91,789 (32.3%)	
	4th level	79,874 (83.2%)	16,101 (16.8%)	95,975 (33.8%)	
	5th level	49,045 (85.6%)	8235 (14.4%)	57,280 (20.2%)	
Dementia	no	103,569 (85.6%)	17,481 (14.4%)	121,050 (42.6%)	0.07 (0.06, 0.08)
	yes	136,575 (83.7%)	26,561 (16.3%)	163,136 (57.4%)	
LTCF type	non-profit	133,411 (84.7%)	24,141 (15.3%)	157,552 (55.4%)	0.02 (0.01–0.03)
	public	10,292 (85.3%)	1775 (14.7%)	12,067 (4.2%)	
	private	96,441 (84.2%)	18,126 (15.8%)	114,567 (40.3%)	
District type	big city	51,999 (83.7%)	10,119 (16.3%)	62,118 (21.9%)	0.05 (0.04, 0.06)
	urban district	80,831 (85.3%)	13,925 (14.7%)	94,756 (33.3%)	
	rural district with dense areas	55,397 (83.8%)	10,744 (16.2%)	66,141 (23.3%)	
	rural district	51,917 (84.9%)	9254 (15.1%)	61,171 (21.5%)	
Districtwide COVID-19 incidence rate	mean (SD)	3139.2 (1256.3)	3645.0 (1374.6)	3217.6 (1288.4)	−0.38 (−0.39, −0.37)
Districtwide COVID-19 incidence rate (SD categories)	<−1 SD	35,258 (91.8%)	3159 (8.2%)	38,417 (13.5%)	0.37 (0.36, 0.38)
	< 0 SD	113,133 (86.7%)	17,288 (13.3%)	130,421 (45.9%)	
	> 0 SD	59,129 (81.8%)	13,150 (18.2%)	72,279 (25.4%)	
	> 1 SD	32,624 (75.7%)	10,445 (24.3%)	43,069 (15.2%)	

*LTCF* Long-term-care facility, *SMD* Standardized Mean Difference, *SD* standard deviation

**Table 2:** Descriptives level 2: Long-term care facilities (*N* = 9869)

Proportion of women (%)	mean (SD)	71.1 (15.6)
Mean Age	mean (SD)	83.6 (4.8)
Mean care dependency level	mean (SD)	3.6 (0.4)
Dementia prevalence (%)	mean (SD)	56.1 (18.4)
LTCF Type	non-profit	5302 (53.7%)
	public	384 (3.9%)
	private	4183 (42.4%)
Number of residents	mean (SD)	72.6 (39.5)
AOK-share	mean (SD)	35.9 (17.8)

*LTCF* Long-term-care facility, *SD* Standard deviation

**Table 3:** Descriptives level 3: districts (*N* = 400)

Proportion of women (%)	mean (SD)	71.8 (3.2)
Mean Age	mean (SD)	84.1 (1.1)
Mean Care dependency level	mean (SD)	3.6 (0.1)
Dementia prevalence (%)	mean (SD)	56.2 (6.6)
LTCF type non-profit (%)	mean (SD)	56.9 (18.8)
LTCF type public (%)	mean (SD)	5.2 (8.8)
LTCF type private (%)	mean (SD)	37.9 (19.5)
Number of residents	mean (SD)	88.6 (18.4)
AOK-share (%)	mean (SD)	39.1 (11.6)
COVID-19 incidence	mean (SD)	3149.6 (1241.3)
District type	big city	67 (16.8%)
	urban district	132 (33.0%)
	rural district with dense areas	100 (25.0%)
	rural district	101 (25.2%)

*LTCF* Long-term-care facility, *SD* Standard deviation, *AOK* AOK Die Gesundheitskasse (General local health and care insurance company)

### Measures

The dependent variable, COVID-19 infection, was determined through a COVID-19 diagnosis (ICD U07.1) by an outpatient physician or through a hospital admission including such diagnosis. In both cases, PCR (polymerase chain reaction) confirmation was not required. Independent variables were measured at the appropriate levels and aggregated for the higher levels. We present these covariates at their basic level of measurement. Individual-level: gender (women/men), age, long-term care dependency level based on the Book XI of the German Social Code (≤ 2–5; highest available record within the study period) and validated dementia diagnosis (based on at least one confirmed inpatient diagnosis or at least two confirmed dementia diagnoses from an outpatient physician in two distinct quarters in 2020); facility level: LTCF type (ownership: non-profit/public/private) and share of AOK-insured residents (we calculated the AOK-share using the number of AOK-insured residents weighted by the length of stay and compared it with the facility size; latest record available 2019); district-level: COVID-19 incidences among the general population [10] (based on the official count of SARS-CoV-2 infections per 100,000 inhabitants within the study period) and an indicator for the territorial type (big city/urban district/rural district with dense areas/rural district) [11]. All metric measures were z-standardized at the level of measurement, namely age, number of residents, AOK-share, and COVID-19 incidence. Furthermore, all aggregate-level measures were z-standardized. For the facility level these were: proportion of women, mean age, mean care dependency level, dementia prevalence; and for the district level: proportion of women, mean age, mean care dependency level, dementia prevalence, proportion of non-profit LTCFs, proportion of public LTCFs, and proportion of private LTCFs, mean number of residents, and mean AOK-share. With the exception of the AOK-share we assume that all covariates included in our analysis are covariates of interest. We use AOK-share as an indicator of the data quality at the LTCF and district level (Fig. 1).

### Statistical analysis

Descriptive statistics of the study population were compared between residents with and without a known COVID-19 infection using standardized mean differences (SMD). In addition, we compared the regional distributions of COVID-19 incidence rates between the general and study populations using z-standardized incidence rates. To describe possible associations between the covariates of interest and COVID-19 infections we applied a three-level multilevel logistic regression. The covariates included in this model were derived from the conceptual framework (Fig. 1), which integrates findings from a systematic review [6] and aligns them with the structural characteristics of German insurance claims data. As further detailed below, the random effects specification was chosen to provide valid statistical inference for the coefficients of interest given the hierarchical data structure while keeping model complexity manageable. We used data measured at the appropriate level and aggregated data for higher levels. Therefore, all categorical variables were transformed into prevalence metrics for higher levels. All metric covariates, including the transformed metrics, were z-standardized and grand mean-centered. This means that individuals within the same facility share identical values for aggregated LTCF covariates, and individuals within the same district share identical values for aggregated district covariates. This facilitates the most straightforward interpretation of contextual effects as coefficient estimates and associated test statistics indicate how much the aggregate effect differs from that at the next lower level and if this difference is statistically significantly different from zero [12, 13]. That is, for covariates measured at the individual level (e.g., age), the facility-level coefficient indicates if the facility-level effect (e.g., facility-average age) differs from the individual-level effect. Further, the district-level coefficient (e.g., district-average age) shows how much the district-level effect differs from the facility-level effect. Similarly, for covariates measured at level 2 (e.g. private ownership, number of residents), the district-level coefficient captures the difference between the district-level effect (e.g., the proportion of privately-owned facilities) from the facility-level effect.

We estimated three models: the Null Model (without covariates), Random Intercept Model I (with covariates at the lowest level), and Random Intercept Model II (including all covariates). The first two models serve primarily as benchmarks for interpreting the full model. For each model we report Marginal R² (variance explained by fixed effects) and Conditional R² (variance explained by fixed plus random effects), as well as the Variance Partition Coefficients (VPC) using the latent response formulation for logistic models with the level-1 variance set to $$\:\raisebox{1ex}{${\pi\:}^{2}$}\!\left/\:\!\raisebox{-1ex}{$3$}\right.$$ [14]. The VPC represents the proportion of latent variance that can be attributed to unmeasured higher-level covariates. Hence for the Null Model without any covariates, the VPC for each level (i.e., facility and district) represents the proportion of the total variance that can be attributed to that level. By comparing the VPC between models without higher-level covariates and with higher-level covariates we can determine how much variance can be attributed to adding these covariates. However, since residual variance is set to $$\:\raisebox{1ex}{${\pi\:}^{2}$}\!\left/\:\!\raisebox{-1ex}{$3$}\right.$$ in logistic regressions we must first introduce the covariates on the lowest level to establish a baseline (Random Intercept Model I). By comparing facility-level and district-level VPCs between models with and without higher-level covariates, we assess how including covariates at those levels changes the share of total latent variance attributable to each upper level.

To explore a possible CLI between number of residents in a LTCF and the COVID-19 incidence we applied a separate model (Fig. 1). Since CLIs require the introduction of the random slope of the lower-level component to avoid overoptimistic statistical inference, we included a random slope for the number of residents [15]. 

For reasons analogous to those affecting CLIs, ‘pure’ lower-level effects can also yield overoptimistic inferences when not accompanied by a random slope [15]. To address this, we conducted sensitivity analyses for all significant lower-level effects by sequentially including one random slope at a time (a model including all random slopes simultaneously would not be estimable due to the large number of random effects variances and covariances). We report below when including a random slope changed the statistical significance of a coefficient.

All analyses were performed in the R environment for statistical computing (R version 4.2.1) and RStudio (Version 2022.07.1).

## Results

**Fig. 2:**
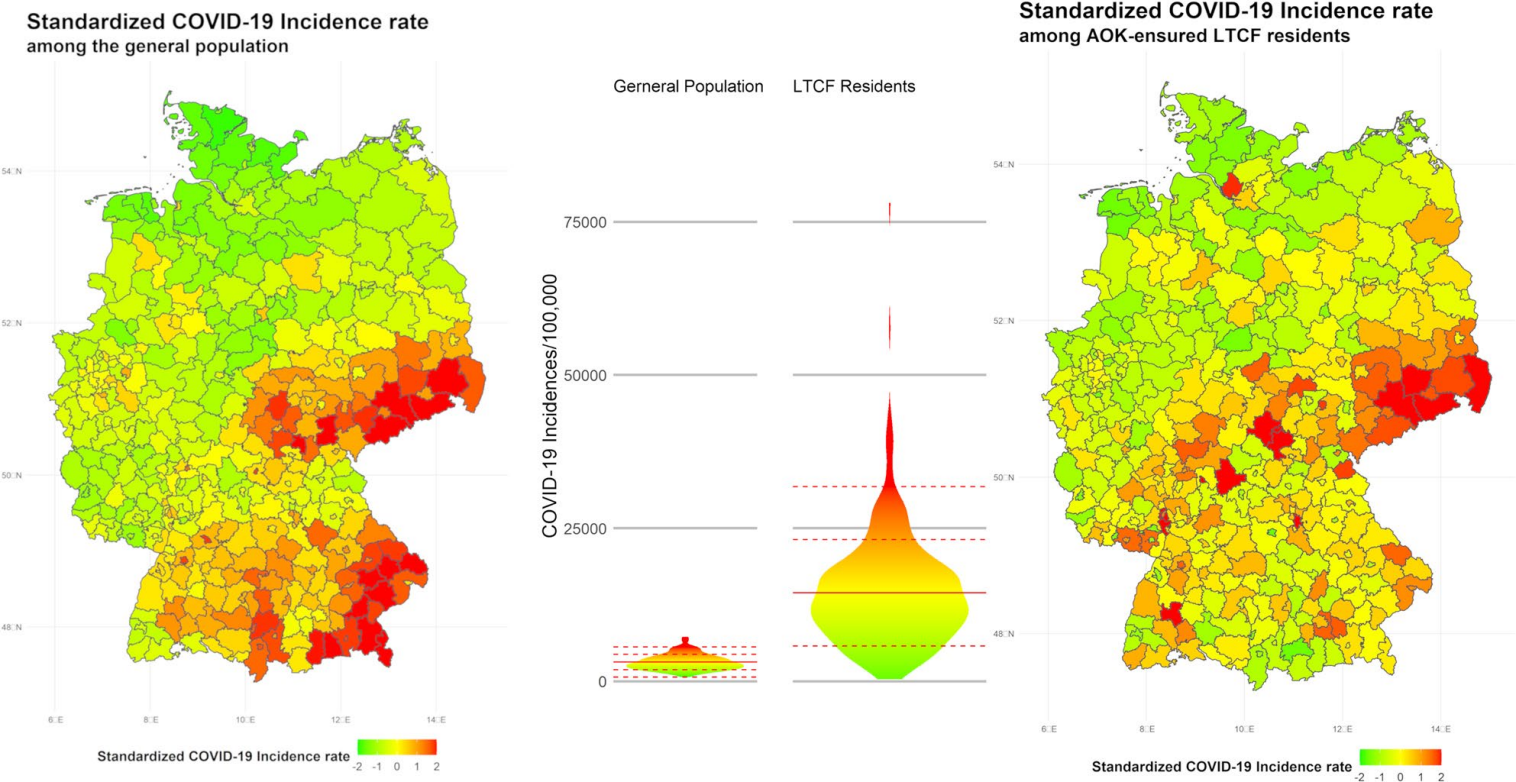
Standardized COVID-19 incidence rate among the general population (left) and AOK-insured LTCF residents (right) on the regional level. Color coding was limited to a standard deviation of ± 2. Values surpassing this range were color-coded according to the nearest limit; Mean COVID-19 incidence rate among the population: 3149.6 per 100,000 inhabitants (SD: 1241.3); Mean COVID-19 incidence rate among AOK-insured LTCF residents: 15497.6 per 100,000 residents (SD: 8697.7). The middle panel shows the distribution of the crude incidence rate per 100,000 people. Red solid line signifies the average district level infection rate, dashed lines the standard deviation

A total of 44,042 (15.5%) COVID-19 infections were recorded among the study population during the study period. The average district-level infection rate among AOK-insured LTCF residents was also 15.5 per 100 residents (SD: 8.7). In the same period, the German government reported almost 2.6 million SARS-CoV-2 infections among the general population. The infections included in our study represent roughly 3.1% of the 2.6 million cases [10]. Fig. 2 shows the distribution of infection rates among the general population and AOK-insured LTCF residents in districts, with higher rates among the general population in southeast Germany (especially Bavaria, Thuringia, and Saxony). For Saxony, but less so for Thuringia and especially Bavaria, this regional clustering is also visible in infection rates among LTCF residents. In addition, there are a few Western German districts with very high LTCF infection rates but without clear regional clustering or correspondingly high infection rates in the general population.

**Fig. 3:**
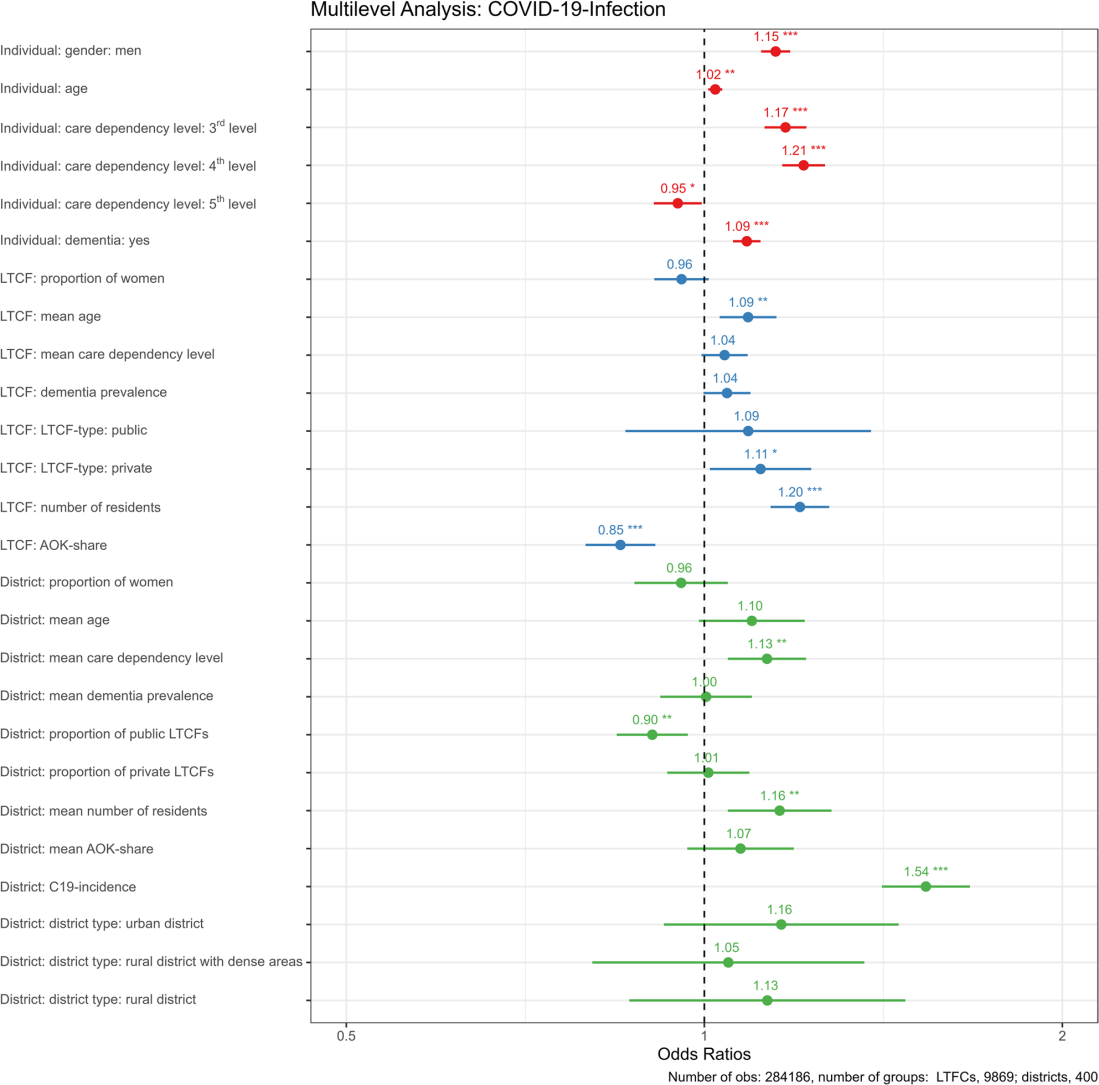
Multilevel logistic regression: SARS-CoV-2 infections among LTCF residents. Number of observations: 284,186, number of groups: 9869 LTCFs, 400 districts

The multilevel regression models of COVID-19 infections among AOK-insured LTCF residents are displayed in Fig. 3; Table 4. Figure 3 illustrates results from the full model (Random Intercept Model II), with Table 4 additionally reporting a null model and a random intercept model with individual-level covariates only.

**Table 4:** Multilevel logistic regression: SARS-COV-2-infection among LTCF-residents

*Covariates*	**Null Model**		**Random Intercept Model I**		**Random Intercept Model II**	
	*Odds Ratios*	*p*	*Odds Ratios*	*p*	*Odds Ratios*	*p*
(Intercept)	0.07 (0.06–0.08)	**<0.001**	0.06 (0.05–0.06)	**<0.001**	0.06 (0.05–0.07)	**<0.001**
Individual level						
Gender: men			1.15 (1.12–1.18)	**<0.001**	1.15 (1.12–1.18)	**<0.001**
Age			1.02 (1.01–1.04)	**0.001**	1.02 (1.01–1.04)	**0.002**
Care dependency level: 3^rd^ level			1.17 (1.12–1.22)	**<0.001**	1.17 (1.12–1.22)	**<0.001**
Care dependency level: 4^th^ level			1.21 (1.16–1.26)	**<0.001**	1.21 (1.16–1.26)	**<0.001**
Care dependency level: 5^th^ level			0.95 (0.91–1.00)	**0.036**	0.95 (0.91–1.00)	**0.030**
Dementia: yes			1.09 (1.06–1.12)	**<0.001**	1.09 (1.06–1.11)	**<0.001**
Facility level						
Proportion of women					0.96 (0.91–1.01)	0.101
Mean age					1.09 (1.03–1.15)	**0.003**
Mean care dependency level					1.04 (0.99–1.09)	0.085
Dementia prevalence					1.04 (1.00–1.09)	0.059
LTCF Type: public					1.09 (0.86–1.38)	0.484
LTCF Type: private					1.11 (1.01–1.23)	**0.030**
Number of residents					1.20 (1.14–1.27)	**<0.001**
AOK-share					0.85 (0.79–0.91)	**<0.001**
District level						
Proportion of women					0.96 (0.87–1.05)	0.331
Mean age					1.10 (0.99–1.21)	0.078
Mean care dependency level					1.13 (1.05–1.22)	**0.002**
Mean Dementia prevalence					1.00 (0.92–1.10)	0.939
Proportion public LTCF					0.90 (0.84–0.97)	**0.004**
Proportion private LTCF					1.01 (0.93–1.09)	0.848
Mean number of residents					1.16 (1.05–1.28)	**0.004**
Mean AOK-share					1.07 (0.97–1.19)	0.183
COVID-19 incidence					1.54 (1.41–1.67)	**<0.001**
District type: urban district					1.16 (0.92–1.46)	0.199
District type: rural district with dense areas					1.05 (0.80–1.36)	0.730
District type: rural district					1.13 (0.86–1.48)	0.371
VPC	0.555		0.554		0.502	
VPC _LTCF_	0.467		0.466		0.465	
VPC _District_	0.088		0.088		0.037	
N _LTCF_	9869		9869		9869	
N _District_	400		400		400	
Observations	284186		284186		284186	
AIC/BIC	188735.7/188767.3		188339.8/188434.8		188029.8/188336.0	
Marginal R^2^/Conditional R^2^	0.000/0.555		0.002/0.557		0.053/0.558	

On the individual level, men had a higher probability of infection (Odds Ratio [OR] 1.15; 95% Confidence Interval [CI] 1.12–1.18). Moreover, higher age (OR 1.02; CI 1.01–1.04) and dementia (OR 1.09; CI 1.06–1.11) were associated with a greater risk. For care dependency level, residents with care dependency level 3 (OR 1.17; CI 1.12–1.22) and 4 (OR 1.21; CI 1.16–1.26) were associated with higher risk of infection compared to the reference group of level ≤ 2. Care dependency level 5 was associated with a lower risk of infection (OR: 0.95 CI 0.91–1.00). However, the sensitivity analyses indicated that age (OR 1.00; CI 0.99–1.02) and care dependency level 5 (OR 0.98; CI 0.93–1.03) were not significantly associated with COVID-19 infections when accompanied with their respective random slope (Supplemental Table 2).

On the facility level, a higher mean age of residents further increased the risk of COVID-19 infections, beyond the individual-level effect of age noted above (OR 1.09; CI 1.03–1.15). Further, we found a trend towards increased risk in LTCFs with higher mean care dependency level (OR 1.04; CI 0.99–1.09) and higher dementia prevalence (OR 1.04; CI 1.00–1.09). However, these were not statistically significant. In addition, the number of residents (OR 1.20; CI 1.14–1.27) showed a positive association with the probability of infection, as did private ownership compared to reference LTCFs with non-profit status (OR 1.11; CI 1.01–1.23). However, private ownership, compared to non-profit LTCFs, did not differ significantly in sensitivity analysis (OR 1.11; CI 1.00–1.23) (Supplemental Table 3).

On the district level, a higher proportion of public LTCFs was associated with lower odds of infection (OR 0.90; CI 0.84–0.97). However, given the prevalence of public LTCFs is fairly low at 5.2% and the standard deviation is 8.8% points the effect size is substantively small and should be interpreted with caution. Further, a greater mean number of residents was associated with a greater risk (OR 1.16; CI 1.05–1.28) and provided an effect, which differed from the facility-level effect. The most pronounced effect was the regional SARS-CoV-2 incidence rate among the general population on the district level (OR 1.54; CI 1.41–1.67). As shown in Tables 4 and 56% of the share of total latent variance is attributable to clustering at the facility level (47%) and the district level (9%). The introduction of higher-level covariates accounted for 58% reduction of the share attributable to the district level (VPC _District_ 0.088 vs. 0.037) but only for 0.2% reduction (VPC _LTCF_ 0.466 vs. 0.465) on the facility level.

We further tested for a potential cross-level interaction between the number of residents on the facility level and the COVID-19 infection rate on the district level but did not find a significant effect (OR 0.99; CI = 0.94–1.04; *p* = 0.709) (Supplemental Table 1).

## Discussion

Our study examined how individual-, facility-, and district-level determinants contribute to the risk of SARS-CoV-2 infections among German residents of LTCFs during the second wave of the COVID-19 pandemic.

At the individual level, our findings showed that infections were significantly associated with gender, dementia, and care dependency level. These results were in line with previous studies, except for a higher risk among male residents in comparison to female residents, which at first glance does not echo the previous findings [6]. But, from the three studies cited by Karimi-Dehkordi et al. only one treats gender as an individual characteristic and that one reports slightly fewer women in the infected population vs. the total population [16]. An additional German study, not included in the review, observed that infection rates reflected the overall gender distribution [4] Our findings may reflect gendered risk-taking behavior, where men are taking higher risks (i.e., refusal to wear masks, social distancing, and other protective measures). Although this explanation is speculative it would align with gendered behavioral patterns towards protective measures documented in previous studies [17, 18]. Likewise, differences between residents with and without dementia may be attributable to lower compliance with protective measures among those with dementia due to their cognitive or mental limitations. Several studies have highlighted the difficulties to ensure protection for this population [19–21]. 

At the facility level, we found associations with residents’ mean age, facility size, and facility type. Previous studies have typically examined age as an individual-level factor, with the exception of Lord et al., who treated it as a contextual factor. These studies found that a higher percentage of residents aged above 65 was associated with lower incidence rates [22]. Our results showed a positive association between mean age and COVID-19 infections distinct from an individual-level effect. There is extensive research on the effect of environmental factors like higher number of beds or occupancy rates on COVID-19 infections which is in line with our finding that residents in larger facilities had a higher risk of infections [23–26]. Furthermore, the effect of the ownership especially the higher risk of residents in privately owned LTCFs is well established in the international literature and mainly discussed in light of larger sizes, lower staffing, and older design standards [27–30]. While our study lacks data to control most of these factors our results are still of interest since it contradicts previous findings of a protective effect of private LTCFs in Germany; thus, our findings appear to be in line with the international evidence [5]. However, since the relative reduction of the VPC is negligible, we must assume that our facility-level factors do not show the whole picture. We refer to the limitations section where we discuss further possible facility-level factors.

At the district level, we found that mean care dependency level, mean facility size and COVID-19 incidences were associated with COVID-19 infections. Among the significantly related factors, the infection rate was the strongest one, which is in line with previous findings, including the two prior German studies [4–6]. Notably, regional population density was not a significant contributor, even though Karimi-Dehkordi et al. cited six studies reporting such an association [6]. 

### Strengths and limitations

The strengths of our study are the large dataset of an underresearched yet vulnerable and hard-to-reach population, the coverage of the claims data, as well as the sophisticated multilevel analytical approach. However, since our study is observational we can only describe associations rather than causal relations. Furthermore, our study has several other limitations, primarily associated with the nature of health insurance claims data in Germany. First, the study period aims to evaluate the second wave of COVID-19 infections in Germany, which took place between 28th September 2020 and 28th February 2021 [9]. Due to the requirements for reporting outpatient care in Germany, our analysis was bound to quarterly data. Therefore, our data includes all outpatient COVID-19 diagnoses between 1 st October 2020 and 31 st March 2021, which also includes part of the third wave. Secondly, our study relies on COVID-19 diagnosis in hospital and outpatient physician settings. While this operationalization allows identification of COVID-19 cases from claims data, the accuracy of ICD coding warrants scrutiny and constitutes a key limitation. On the one hand, this could lead to an overestimation since our study did not require confirmation of this diagnosis with PCR testing, as such data was not available. Since claims data indicates only that people were tested but not the results of the testing, the added value of including a PCR testing requirement is limited. On the other hand, it is possible that COVID-19 infections did not get recognized or were not reported, e.g., due to a lack of symptoms or the challenging circumstances in the LTCFs at that time which could lead to an underestimation. Our data were nonetheless compared to the German federal statistics on COVID-19 cases [3]. While we found more COVID-19 cases in the claims data than we would expect from the national statistics for the second quarter of 2020 corresponding with the first wave of the COVID-19 pandemic, we did not find systematic differences for the study period. Hence, we assume our data is at least comparable to other available data sources in Germany. Further, the vaccination program against the SARS-CoV-2 virus started within the study period following the conditional market authorization on 21 st December 2020, and residents of LTCF were included in the first group of eligible people in Germany [31]. Until the end of our study period, over 9.4 million people (11.3% of the German population) had been vaccinated at least once, and almost half of them twice [3]. However, due to the federal funding of the vaccination program, we do not have any record of the vaccination status of LTCF residents. Therefore, we cannot control for any preventive effect on infections among the study population, as was reported in other countries [32–34], nor for possible biases arising from facility- and/or district-level differences in the starting date or speed of vaccination rollout. In addition, due to data limitations, our study cannot account for any LTCF-level factors other than the number of residents and ownership. In particular, we were not able to consider factors like building type or age, room type, crowdedness, or other design elements of the facilities, which were reported in other studies [27, 35, 36]. The same applies to performance and management factors like infection control policy and pandemic preparedness, facility ratings, and caregiver-to-resident ratio [37–39]. This may also explain the small relative reduction in the facility-level VPC achieved by our covariates. Finally, it should be highlighted, that the factors of age, dementia, and level of care dependency may be interconnected through complex causal pathways, which to test where beyond the scope of the present study (see Fig. 1).

### Implications for research, policy, and practice

Across multiple levels, we demonstrated that the risk of COVID-19 infections was differentially associated with a variety of individual-, facility- and district-level factors. Future measures to tackle infectious diseases in endemic or pandemic situations should take an evidence-informed orchestrated multilevel approach.

As expected, dementia was associated with COVID-19 infections. As this characteristic is also associated with poorer infection outcomes, our findings underline the need to protect these particularly vulnerable populations. However, we also find some evidence that infections might be associated with the mobility pattern and risk-taking behavior of LTCF residents, suggesting that measures to prevent infection should be tailored to take these factors into account. Further, we found that the regional incidence did predict COVID-19 infections among residents from LTCFs. This finding underscores the fact that the protection of LTCF residents cannot established solely in the facilities. The reduction of regional incidences will be useful to protect LTCF residents.

Lastly, while our analysis uncovered several facility-level factors of infection risks, the latter were mostly aggregated variables based on resident characteristics. Due to data limitations, we were not able to study the role of facility-level factors that would be more amenable to direct modification. Our facility-level factors, in addition to in literature established facility-level factors such as staffing, crowding, facility- and room design could have direct implications for public health policies including visitation policies, screening strategies. Yet, more fine-grained data are needed to draw these conclusions. A database on the characteristics of German LTCFs should be set up to enable further research into facility characteristics and how they relate with infection rates. This database, ideally, should be linkable to both national statistics on infectious diseases and routine health insurance data to provide a future framework for evaluating protective measures.

## Conclusion

We presented a multilevel approach to examining COVID-19 infections among LTCF residents. We found that male sex, dementia, and medium (as opposed to low) care dependency were important individual-level factors for an elevated infection risk. On the facility level, a higher mean age among residents and a higher number of residents stood out as important contextual risk factors. Finally, on the district level, a higher proportion of public LTCFs was associated with a lower, while a higher general population incidence rate was associated with a higher risk of infections. These findings not only contribute to a better understanding of infection dynamics in LTCF during the COVID-19 pandemic; they also suggest important lessons for preventive measures during future pandemics as well as seasonal outbreaks of infectious diseases.

## Supplementary Information

Supplementary Material 1

## Data Availability

While access to the data statutory health insurance funds for research purposes is possible under German Social Law (SGB V § 287) the data can only be made available when certain data-protecting measurements are met. For assistance regarding data access please contact the first author or wido@wido.bv.aok.de.
