# Supplementary material for: COVID-19 infections in German long-term care facilities: a descriptive three-level analysis using claims and infection statistics data from October 2020 to March 2021
Source: BMC Public Health. 2026 Feb 7;26:717. doi: 10.1186/s12889-026-26510-5 (PMC12930546; doi:10.1186/s12889-026-26510-5)
Supplement: Supplementary file 1 — Supplementary Material 1 [file 12889_2026_26510_MOESM1_ESM.pdf]

Supplemental Table 1 Multilevel logistic regression: SARS-COV-2-infection among LTCF-residents with cross-level interaction between number of residents and COVID-19 infection rates

	**Cross Level Interaction**	
Covariates	*Odds Ratios*	*p*
(Intercept)	0.05 (0.03 – 0.06)	**<0.001**
**Individual level**		
Gender: men	1.15 (1.12 – 1.18)	**<0.001**
Age	1.02 (1.01 – 1.04)	**0.002**
Care dependency level: 3rd level	1.17 (1.12 – 1.22)	**<0.001**
Care dependency level: 4th level	1.21 (1.16 – 1.26)	**<0.001**
Care dependency level: 5th level	0.95 (0.91 – 1.00)	**0.030**
Dementia: yes	1.09 (1.06 – 1.11)	**<0.001**
**Facility level**		
Proportion of women	0.96 (0.91 – 1.01)	0.096
Mean age	1.09 (1.03 – 1.15)	**0.003**
Mean care dependency level	1.04 (0.99 – 1.09)	0.082
Dementia prevalence	1.05 (1.00 – 1.09)	0.057
LTCF Type: public	1.09 (0.86 – 1.38)	0.490
LTCF Type: private	1.11 (1.01 – 1.23)	**0.030**
Number of residents	1.20 (1.13 – 1.27)	**<0.001**
AOK-share	0.85 (0.79 – 0.91)	**<0.001**
**District level**		
Proportion of women	0.95 (0.87 – 1.04)	0.310
Mean age	1.10 (0.99 – 1.21)	0.078
Mean care dependency level	1.13 (1.05 – 1.22)	**0.001**
Mean Dementia prevalence	1.01 (0.92 – 1.10)	0.848
Proportion public LTCF	0.90 (0.84 – 0.97)	**0.004**
Proportion private LTCF	1.01 (0.93 – 1.09)	0.893
Mean number of residents	1.16 (1.05 – 1.28)	**0.004**
Mean AOK-share	1.01 (1.00 – 1.01)	0.159

COVID-19 incidence	1.53 (1.40 – 1.67)	**<0.001**
District type: urban district	1.16 (0.92 – 1.45)	0.215
District type: rural district with dense areas	1.04 (0.80 – 1.35)	0.770
District type: rural district	1.12 (0.86 – 1.46)	0.406
**Cross Level Interaction**		
Number of residents * COVID-19 incidence	0.99 (0.94 – 1.04)	0.709
**District Level Random Slope**		
Number of residents	0.00	
VPC	0.502	
VPC_{LTCF}	0.457	
$VPC_{District}$	0.045	
N_{LTCF}	9869	
$N_{District}$	400	
Observations	284186	
AIC / BIC	188033.6 / 188371.4	
Marginal R^2 / Conditional R^2	0.106 / NA	

Supplemental Table 2: Sensitivity analyses multilevel logistic regression: SARS-COV-2-infection among LTCF-residents with random slopes for significant individual level covariates

	Random Slope Model Gender		Random Slope Model Age		Random Slope Model Care Dependency Level		Random Slope Model Dementia	
Covariates	*Odds Ratios*	*p*	*Odds Ratios*	*p*	*Odds Ratios*	*p*	*Odds Ratios*	*p*
(Intercept)	0.04 (0.03 – 0.06)	**<0.001**	0.05 (0.03 – 0.06)	**<0.001**	0.05 (0.03 – 0.06)	**<0.001**	0.05 (0.03 – 0.07)	**<0.001**
**Individual level**								
Gender: men	1.22 (1.18 – 1.25)	**<0.001**	1.15 (1.12 – 1.18)	**<0.001**	1.15 (1.12 – 1.18)	**<0.001**	1.15 (1.12 – 1.18)	**<0.001**
Age	1.02 (1.01 – 1.04)	**0.002**	1.0 (0.99 – 1.02)	0.524	1.02 (1.01 – 1.04)	**0.004**	1.02 (1.01 – 1.03)	**0.003**
Care dependency level: 3rd level	1.17 (1.12 – 1.22)	**<0.001**	1.17 (1.12 – 1.22)	**<0.001**	1.16 (1.12 – 1.21)	**<0.001**	1.17 (1.12 – 1.22)	**<0.001**
Care dependency level: 4th level	1.21 (1.16 – 1.26)	**<0.001**	1.21 (1.16 – 1.26)	**<0.001**	1.22 (1.16 – 1.27)	**<0.001**	1.21 (1.16 – 1.26)	**<0.001**
Care dependency level: 5th level	0.95 (0.91 – 0.99)	**0.029**	0.95 (0.91 – 0.99)	**0.029**	0.98 (0.93 – 1.03)	0.426	0.95 (0.91 – 0.99)	**0.028**
Dementia: yes	1.09 (1.06 – 1.11)	**<0.001**	1.09 (1.06 – 1.11)	**<0.001**	1.09 (1.06 – 1.12)	**<0.001**	1.05 (1.02 – 1.08)	**<0.001**
**Facility level**								
Proportion of women	0.96 (0.91 – 1.01)	0.132	0.96 (0.91 – 1.01)	0.117	0.96 (0.91 – 1.01)	0.095	0.96 (0.91 – 1.01)	0.114
Mean age	1.09 (1.04 – 1.16)	**0.001**	1.10 (1.04 – 1.16)	**0.001**	1.09 (1.03 – 1.15)	**0.003**	1.09 (1.03 – 1.15)	**0.002**
Mean care dependency level	1.04 (1.00 – 1.09)	0.069	1.04 (1.00 – 1.09)	0.066	1.04 (0.99 – 1.09)	0.090	1.04 (0.99 – 1.09)	0.114
Dementia prevalence	1.04 (1.00 – 1.09)	0.062	1.05 (1.00 – 1.09)	0.055	1.04 (1.00 – 1.09)	0.060	1.05 (1.00 – 1.10)	0.048
LTCF Type: public	1.08 (0.85 – 1.36)	0.542	1.07 (0.85 – 1.36)	0.557	1.09 (0.86 – 1.38)	0.483	1.09 (0.86 – 1.38)	0.482
LTCF Type: private	1.12 (1.01 – 1.23)	**0.028**	1.11 (1.01 – 1.23)	**0.032**	1.12 (1.01 – 1.23)	**0.028**	1.11 (1.01 – 1.23)	**0.030**
Number of residents	1.20 (1.13 – 1.27)	**<0.001**	1.20 (1.13 – 1.27)	**<0.001**	1.21 (1.14 – 1.28)	**<0.001**	1.20 (1.13 – 1.27)	**<0.001**

AOK-share	0.85 (0.79 – 0.91)	**<0.001**	0.85 (0.79 – 0.91)	**<0.001**	0.85 (0.79 – 0.91)	**<0.001**	0.85 (0.80 – 0.91)	**<0.001**
**District level**								
Proportion of women	0.96 (0.87 – 1.04)	0.351	0.96 (0.87 – 1.05)	0.322	0.96 (0.87 – 1.05)	0.331	0.96 (0.87 – 1.05)	0.330
Mean age	1.10 (0.99 – 1.22)	0.076	1.10 (0.99 – 1.22)	0.076	1.10 (0.99 – 1.21)	0.079	1.10 (0.99 – 1.22)	0.073
Mean care dependency level	1.13 (1.05 – 1.22)	**0.002**	1.13 (1.05 – 1.22)	**0.002**	1.13 (1.05 – 1.22)	**0.002**	1.13 (1.05 – 1.22)	**0.002**
Mean Dementia prevalence	1.00 (0.92 – 1.10)	0.971	1.00 (0.92 – 1.10)	0.944	1.00 (0.92 – 1.10)	0.93	1.00 (0.92 – 1.09)	0.972
Proportion public LTCF	0.91 (0.84 – 0.97)	**0.005**	0.90 (0.84 – 0.97)	**0.004**	0.90 (0.84 – 0.97)	**0.004**	0.90 (0.84 – 0.97)	**0.004**
Proportion private LTCF	1.01 (0.93 – 1.09)	0.829	1.01 (0.93 – 1.09)	0.856	1.01 (0.93 – 1.09)	0.818	1.01 (0.93 – 1.09)	0.865
Mean number of residents	1.16 (1.05 – 1.28)	**0.004**	1.16 (1.05 – 1.28)	**0.005**	1.16 (1.05 – 1.28)	**0.004**	1.16 (1.05 – 1.28)	**0.005**
Mean AOK-share	1.01 (1.00 – 1.01)	0.145	1.01 (1.00 – 1.01)	0.168	1.01 (1.00 – 1.01)	0.190	1.01 (1.00 – 1.01)	0.193
COVID-19 incidence	1.53 (1.41 – 1.6)	**<0.001**	1.54 (1.41 – 1.67)	**<0.001**	1.53 (1.41 – 1.67)	**<0.001**	1.54 (1.41 – 1.67)	**<0.001**
District type: urban district	1.16 (0.92 – 1.45)	0.208	1.16 (0.92 – 1.45)	0.208	1.16 (0.92 – 1.46)	0.202	1.16 (0.92 – 1.46)	0.197
District type: rural district with dense areas	1.054 (0.80 – 1.36)	0.753	1.05 (0.80 – 1.36)	0.744	1.05 (0.81 – 1.37)	0.707	1.0 (0.81 – 1.37)	0.712
District type: rural district	1.13 (0.87 – 1.48)	0.356	1.13 (0.87 – 1.48)	0.365	1.13 (0.86 – 1.47)	0.381	1.13 (0.87 – 1.48)	0.358
Random Slope $_{LTCF}$	Gender: men 0.03		Age 0.01		Care dependency level: 3rd level 0.01<br>Care dependency level: 4th level 0.14<br>Care dependency level: 5th level 0.19		Dementia: yes 0.08	
VPC	0.496		0.501		0.429		0.493	
VPC $_{LTCF}$	0.465		0.466		0.415		0.464	

VPC $_{District}$	0.031	0.035	0.014	0.029
N $_{LTCF}$	9869	9869	9869	9869
N $_{District}$	400	400	400	400
Observations	284186	284186	284186	284186
AIC / BIC	188010.6 / 188337.9	188025.0 / 188352.3	187995.2 / 188396.4	187997.6 / 188324.9
Marginal R^2 / Conditional R^2	0.053 / 0.560	0.053 / 0.559	0.106 / NA	0.052 / 0.560

Supplemental Table 3: Sensitivity analyses multilevel logistic regression: SARS-COV-2-infection among LTCF-residents with random slopes for significant facility level covariates

	**Random Slope Model Facility Level: Mean Age**		**Random Slope Model Facility Level: LTCF Type**		**Random Slope Model Facility Level: Number of Residents**	
Covariates	*Odds Ratios*	*p*	*Odds Ratios*	*p*	*Odds Ratios*	*p*
(Intercept)	0.05 (0.03 – 0.06)	**<0.001**	0.05 (0.03 – 0.07)	**<0.001**	0.05 (0.03 – 0.06)	**<0.001**
**Individual level**						
Gender: men	1.15 (1.12 – 1.18)	**<0.001**	1.15 (1.12 – 1.18)	**<0.001**	1.15 (1.12 – 1.18)	**<0.001**
Age	1.02 (1.01 – 1.04)	**0.002**	1.02 (1.01 – 1.04)	0.002	1.02 (1.01 – 1.04)	**0.002**
Care dependency level: 3rd level	1.17 (1.12 – 1.22)	**<0.001**	1.17 (1.12 – 1.22)	**<0.001**	1.17 (1.12 – 1.22)	**<0.001**
Care dependency level: 4th level	1.21 (1.16 – 1.26)	**<0.001**	1.21 (1.16 – 1.26)	**<0.001**	1.21 (1.16 – 1.26)	**<0.001**
Care dependency level: 5th level	0.95 (0.91 – 0.99)	**0.030**	0.95 (0.91 – 0.99)	**0.030**	0.95 (0.91 – 1.00)	**0.030**
Dementia: yes	1.09 (1.06 – 1.11)	**<0.001**	1.09 (1.06 – 1.11)	**<0.001**	1.09 (1.06 – 1.11)	**<0.001**
**Facility level**						
Proportion of women	0.96 (0.91 – 1.01)	0.101	0.96 (0.91 – 1.01)	0.108	0.96 (0.91 – 1.01)	0.097
Mean age	1.09 (1.03 – 1.15)	**0.002**	1.09 (1.03 – 1.15)	**0.003**	1.09 (1.03 – 1.15)	**0.003**
Mean care dependency level	1.04 (0.99 – 1.09)	0.095	1.04 (1.00 – 1.09)	0.085	1.04 (1.00 – 1.09)	0.082
Dementia prevalence	1.05 (1.00 – 1.09)	0.056	1.04 (1.00 – 1.09)	0.063	1.04 (1.00 – 1.09)	0.058
LTCF Type: public	1.09 (0.86 – 1.38)	0.482	1.08 (0.84 – 1.39)	0.543	1.09 (0.86 – 1.38)	0.491
LTCF Type: private	1.11 (1.01 – 1.23)	**0.031**	1.11 (1.00 – 1.23)	0.057	1.11 (1.01 – 1.23)	**0.030**
Number of residents	1.20 (1.14 – 1.27)	**<0.001**	1.20 (1.14 – 1.27)	**<0.001**	1.20 (1.13 – 1.27)	**<0.001**
AOK-share	0.85 (0.79 – 0.91)	**<0.001**	0.85 (0.80 – 0.91)	**<0.001**	0.85 (0.79 – 0.91)	**<0.001**
**District level**						

Proportion of women	0.95 (0.87 – 1.05)	0.315	0.95 (0.87 – 1.04)	0.300	0.95 (0.87 – 1.04)	0.331
Mean age	1.10 (0.99 – 1.22)	0.075	1.10 (0.99 – 1.22)	0.064	1.10 (0.99 – 1.22)	0.077
Mean care dependency level	1.13 (1.05 – 1.22)	**0.001**	1.13 (1.05 – 1.22)	**0.001**	1.13 (1.05 – 1.22)	**0.001**
Mean Dementia prevalence	1.00 (0.92 – 1.10)	0.981	1.00 (0.92 – 1.09)	0.956	1.01 (0.92 – 1.10)	0.853
Proportion public LTCF	0.90 (0.84 – 0.97)	**0.004**	0.90 (0.84 – 0.97)	**0.004**	0.90 (0.84 – 0.97)	**0.004**
Proportion private LTCF	1.01 (0.93 – 1.09)	0.831	1.01 (0.93 – 1.09)	0.810	1.01 (0.93 – 1.09)	0.898
Mean number of residents	1.16 (1.05 – 1.28)	**0.004**	1.16 (1.05 – 1.28)	**0.004**	1.16 (1.05 – 1.28)	**0.004**
Mean AOK-share	1.01 (1.00 – 1.01)	0.185	1.01 (1.00 – 1.01)	0.220	1.01 (1.00 – 1.01)	0.157
COVID-19 incidence	1.54 (1.41 – 1.67)	**<0.001**	1.54 (1.41 – 1.68)	**<0.001**	1.53 (1.41 – 1.67)	**<0.001**
District type: urban district	1.17 (0.93 – 1.46)	0.187	1.15 (0.92 – 1.68)	0.219	1.16 (0.92 – 1.45)	0.212
District type: rural district with dense areas	1.06 (0.81 – 1.37)	0.684	1.06 (0.81 – 1.38)	0.672	1.04 (0.80 – 1.36)	0.766
District type: rural district	1.13 (0.87 – 1.48)	0.357	1.13 (0.87 – 1.48)	0.352	1.12 (0.86 – 1.48)	0.404
Random Slope $_{District}$	Mean Age<br>0.00		LTCF Type: public<br>0.29<br><br>LTCF Type: private<br>0.09		Number of residents<br>0.00	
VPC	0.501		0.486		0.502	
VPC $_{LTCF}$	0.456		0.318		0.457	
VPC $_{District}$	0.045		0.168		0.045	
N $_{LTCF}$	9869		9869		9869	
N $_{District}$	400		400		400	
Observations	284186		284186		284186	
AIC / BIC	188033.2 / 188360.5		188034.3 / 188393.3		188032.4 / 188359.7	
Marginal R^2 / Conditional R^2	0.053 / 0.558		0.053 / 0.559		0.107 / NA	
